# Effects of Low-Voltage Electrostatic Field Combined with Modified Atmosphere Packaging on Postharvest Quality and Senescence of Chinese Flowering Cabbage

**DOI:** 10.3390/foods15101674

**Published:** 2026-05-11

**Authors:** Yaqi Li, Yulong Chen, Fanwei Dai, Hua Huang, Zhaoqi Zhang, Fang Fang, Ling Wang

**Affiliations:** 1 College of Horticulture, South China Agricultural University, Guangzhou 510642, China; liyaqi061@163.com (Y.L.);; 2 Sericultural & Agri-Food Research Institute Guangdong Academy of Agricultural Sciences, Key Laboratory of Functional Foods, Ministry of Agriculture and Rural Affairs, Guangdong Key Laboratory of Agricultural Products Processing, Guangzhou 510610, China; 3 Institute of Fruit Tree Research, Guangdong Academy of Agricultural Sciences, Key Laboratory of South Subtropical Fruit Biology and Genetic Resource Utilization, Ministry of Agriculture and Rural Affairs, Guangdong Provincial Key Laboratory of Tropical and Subtropical Fruit Tree Research, Guangzhou 510640, China

**Keywords:** Chinese flowering cabbage, low-voltage electrostatic field (LVEF), modified atmosphere packaging (MAP), stem hollowing, reactive oxygen species (ROS)

## Abstract

Postharvest senescence and stem hollowing severely compromise the commercial value and edible quality of Chinese flowering cabbage. The synergistic effects of a low-voltage electrostatic field (LVEF) and modified atmosphere packaging (MAP) on mitigating postharvest deterioration in Chinese flowering cabbage was elucidated. The results demonstrated that the combined LVEF+MAP treatment significantly outperformed individual treatments, effectively delaying senescence, reducing weight loss, suppressing stem hollowing, and preserving nutritional components such as Vitamin C and soluble protein. Physiologically, the combined treatment alleviated oxidative stress by inhibiting the accumulation of reactive oxygen species (ROS; O_2_^•−^ and H_2_O_2_) and reducing malondialdehyde (MDA) content. This protective effect was driven by maintaining the activities of antioxidant enzymes, including superoxide dismutase (SOD), peroxidase (POD), and catalase (CAT). Gene expression analyses further revealed that LVEF+MAP upregulated the expression of antioxidant-related genes (*BrSOD*, *BrCAT* and *BrPOD*) and modulated ROS generation-related genes (*BrRbohC* and *BrRbohD*), suggesting active regulation of ROS homeostasis at the transcriptional level. Furthermore, principal component analysis (PCA) and correlation networks confirmed a coordinated defense response, with the LVEF+MAP group consistently maintaining the highest comprehensive quality scores during storage. Overall, the LVEF+MAP combination provided a favorable external microenvironment and activated internal defense signaling to maximize postharvest preservation of Chinese flowering cabbage, extending the commercially acceptable shelf life to 12 days under the tested conditions.

## 1. Introduction

Chinese flowering cabbage (*Brassica rapa* ssp. *parachinensis*) is an annual or biennial vegetable belonging to the *Brassicaceae* family [1]. It is prized for its tender texture, sweet flavor, and high nutritional value; notably, its stems, leaves, and flowers are all edible [2,3]. However, Chinese flowering cabbage undergoes rapid postharvest quality deterioration because of its intense physiological and metabolic activity, with commercial losses reported to range from 20% to 40% [4]. In addition, the basal cutting process during harvest often causes substantial mechanical injury. This makes the stem tissues more prone to lignification, pith whitening, and hollowing during subsequent storage and transportation, thereby reducing eating quality and market value [5]. Several preservation approaches have been applied to delay postharvest senescence in Chinese flowering cabbage. Modified atmosphere packaging (MAP) effectively preserves Chinese flowering cabbage at 20 °C, extending its storage period to 7 days [6]. Exogenous melatonin maintains the leaf quality of postharvest Chinese flowering cabbage by modulating respiratory metabolism and energy status, thereby extending its storage period to 7 days at 15 °C [7]. CPPU treatment significantly delayed the yellowing of Chinese flowering cabbage at 4 °C by attenuating oxidative damage, maintaining unsaturated fatty acid levels and membrane integrity, and markedly delaying energy deficiency, thereby extending its storage period to 20 days [8]. However, a single preservation method is insufficient to prevent postharvest quality deterioration in Chinese flowering cabbage, prompting the exploration of various techniques to delay senescence.

As an innovative physical preservation method, electrostatic field technology is non-thermal, low-energy, eco-friendly, and cost-effective, and is classified by output voltage into high-voltage (>2.5 kV, HVEF) and low-voltage (≤2.5 kV, LVEF) types [9,10]. An LVEF generates a negatively ionized environment and exerts ion-mediated effects. Specifically, it inhibits microbial respiration and proliferation, regulates tissue enzyme activities, and reduces water migration, thereby delaying spoilage and maintaining product quality [10]. In strawberry, an LVEF reduced weight loss, decay incidence, and quality deterioration during storage at 4 ± 1 °C for 10 days by suppressing ROS and MDA, enhancing antioxidant activity, and maintaining ascorbic acid and glutathione [11]. In litchi, an LVEF inhibited pericarp browning, preserved soluble solids, ascorbic acid, and soluble protein contents, and maintained fruit quality during storage under low-temperature conditions [12]. In fresh-cut pineapple, an LVEF decreased respiration rate and browning, inhibited polyphenol oxidase-related activities, and enhanced antioxidant capacity, thereby reducing membrane lipid peroxidation and preserving membrane integrity, and improved quality maintenance during 12 days of cold storage [13]. An LVEF has been reported to regulate enzyme activities and metabolic processes, inhibit cell wall degradation and lignification, and preserve aroma and nutritional quality in fresh bamboo shoots and fresh-cut broccoli [14,15]. In addition, combining an LVEF with chili pepper leaf essential oil (CLEO) synergistically improves the postharvest quality of chili peppers by reducing weight loss and malondialdehyde, while enhancing firmness, color, antioxidant capacity, and cell wall stability [16]. Collectively, these studies indicate that an LVEF has considerable potential to delay postharvest senescence, regulate cell wall metabolism, and maintain the nutritional and sensory quality of fruit and vegetables. However, the preservation efficacy of an LVEF applied alone remains limited in some cases and may vary substantially among different commodities [17].

Modified atmosphere packaging (MAP) is one of the most widely used technologies for preserving postharvest quality in horticultural crops [18]. MAP regulates the gaseous microenvironment surrounding the product, thereby suppressing physiological activity and microbial growth, reducing water loss, and extending shelf life [19]. Due to its convenience and cost-effectiveness, it is often combined with other preservation techniques to enhance postharvest quality [20,21]. Recent studies have increasingly focused on combining electrostatic field treatment with MAP, and this integrated strategy has demonstrated clear advantages in enhancing postharvest quality and extending storage duration, such as prolonging the storage life of cherry tomatoes up to 24 days at 10 °C [22], and extending the shelf life of button mushrooms from 6 days to over 12 days at 4 °C [23]. MAP provides a favorable internal atmosphere that reduces metabolic activity, while a low-voltage electrostatic field (LVEF) may further regulate physiological metabolism and oxidative status. Consequently, the two treatments may exert synergistic effects in maintaining sensory quality, nutritional attributes, and antioxidant capacity, thereby improving postharvest quality retention and extending storage life. However, the combined application of an LVEF and MAP in Chinese flowering cabbage has been rarely explored, especially with respect to stem quality maintenance and the associated physiological and molecular regulatory mechanisms.

The present study aimed to investigate the effects of a perforated food storage bag (CK), modified atmosphere packaging (MAP), a low-voltage electrostatic field (LVEF), and their combined treatment (LVEF+MAP) on the postharvest quality of ‘Ningxia’ Chinese flowering cabbage during storage, with particular emphasis on stem quality maintenance. To clarify the individual and synergistic effects of the LVEF and MAP, we evaluated visual quality, nutritional attributes, ROS metabolism, activities of antioxidant enzyme, and the expression of their related genes. This study is expected to provide a scientific basis for the development of green postharvest preservation strategies for Chinese flowering cabbage and to support the broader application of an LVEF in vegetables.

## 2. Materials and Methods

### 2.1. Plant Materials and Treatments

Chinese flowering cabbages (*Brassica rapa* ssp. *parachinensis*) with tender stem and green leaves were harvested from a commercial farm near Guangzhou about 35 d after seeding. Chinese flowering cabbage was at the flush-cut stage (stalk level with leaf tips, flowers about to open), when the main stalk stands slightly above leaves with 1~2 open flowers. The stalks were cut 2~3 nodes above the base, yielding an 18~20 cm length and 1.5~2 cm cut diameter, then immediately transported to the laboratory. Samples characterized by uniform size, shape, and color without damages were selected for the study. No washing, trimming, or additional cleaning procedures were applied prior to packaging. The cabbages were randomly divided into four treatment groups. For the control (CK) and LVEF treatments, samples were packed in conventional polyethylene (PE) bags (thickness 30 μm, perforated PE bags). For the MAP and LVEF+MAP treatments, samples were sealed in gas-selective modified atmosphere packaging (MP30; thickness 30 μm, O_2_ permeability 5625 cm^3^·m^−2^·d^−1^·atm^−1^, and water vapor transmission rate 17.812 g·m^−2^·d^−1^). This is a passive MAP system. Each package contained approximately 280 g of cabbage in bags (35 cm × 30 cm) and was hermetically sealed using a manual impulse sealer (PFS-300; Tell Packaging Machinery Co., Ltd., Shanghai, China). All samples were stored at 15 ± 1 °C and 75% relative humidity. The storage temperature (15 ± 1 °C) and relative humidity (75%) were chosen to simulate typical commercial shelf-life conditions and to accelerate the senescence process for evaluation purposes, consistent with established postharvest research methodologies for this species [5,6,24]. The LVEF and LVEF+MAP groups were subjected to a 1 kV electrostatic field for 1 h every 3 d in an electromagnetic coupling constant-temperature chamber (MEF10, Induce, Wuxi, China), equipped with built-in electrodes that generated a stable and uniformly distributed electrostatic field within the chamber, allowing for consistent exposure of the samples placed inside. The electrostatic field intensity was selected based on previous studies and preliminary trials (Supplementary Figure S1), which indicated that 1 kV was effective in maintaining postharvest quality, whereas excessively high voltages may cause physiological damage to plant tissues. Sampling was conducted on days 0, 3, 6, 9 and 12, with three independent replicates (bags) per treatment at each time point. At each sampling interval, the second and third leaves from the bottom, along with the bolting stem section (between the first and third nodes), were collected from 10 individual plants per treatment. During storage, the visual appearance of samples was documented using a high-resolution digital camera under consistent laboratory lighting and a fixed focal distance to ensure comparability of the photographic evidence presented. All samples were immediately frozen in liquid nitrogen and stored at −80 °C for further biochemical and molecular analysis.

### 2.2. Determination of Weight Loss

Weight loss was determined using a standard gravimetric method [11]. Three specific packages from each of the four treatment groups were permanently marked, and their initial fresh weights were recorded on day 0. These identical packages were subsequently weighed on each designated sampling day. Weight loss was expressed as a percentage (%), calculated as the reduction in weight relative to the initial weight.

### 2.3. Chlorophyll Content Analysis

Chlorophyll content was evaluated following Zhang et al. (2011) [24]. Fresh leaf tissues (0.5 g) were thoroughly ground using liquid nitrogen, followed by extraction in 5 mL of a cold acetone–ethanol mixture (2:1, *v*/*v*). This process was carried out at 4 °C for 12 h under dark conditions. Subsequently, the mixture was centrifuged to collect the supernatant, and the extraction was continued until the resulting residue appeared completely colorless. Spectrophotometric readings were then obtained at 645 and 663 nm. Chlorophyll content was indicated as mg kg^−1^ on the basis of fresh weight.

### 2.4. Analysis of In-Package Gas Composition

Headspace O_2_ and CO_2_ levels in the bags were determined using a gas analyzer (CheckMate 3, Dansensor, Ringsted, Denmark) and expressed as percentages (%). Self-sealing rubber pads were affixed to the left, right, and upper-center areas of each package to facilitate needle penetration without gas leakage. Measurements were performed sequentially at these three positions for each of the three replicate bags per treatment. The final concentrations were expressed as the mean values of these triplicate measurements.

### 2.5. Determination of Soluble Protein Content

Protein content was measured via the Coomassie Brilliant Blue method [25]. Briefly, 0.5 g of frozen tissue was homogenized in 5 mL of deionized water. The homogenate was centrifuged at 12,000× *g* for 20 min at 4 °C. Thereafter, 1 mL of the clear extract was mixed with 5 mL of the protein-binding reagent and incubated for 2 min before measuring the absorbance at 595 nm. The results were expressed as mg g^−1^ on the basis of fresh weight.

### 2.6. Determination of Vitamin C Content

The content of Vitamin C (Vc) was quantified through a fluorometric approach based on the procedure described by Roy et al. (1976) [26] with slight adjustments. Briefly, 0.5 g of frozen stem tissue was homogenized with 5 mL of 2% (*w*/*v*) oxalic acid, followed by mixing with 0.2 g of activated carbon in light-shielded (brown) tubes. The mixture was vortexed thoroughly and centrifuged using 8000× *g* for 10 min under 4 °C. Subsequently, 1 mL of the oxidized supernatant was transferred into two separate tubes, with tube A serving as the sample and tube B as the blank. Tube A received 1 mL of sodium acetate (250 g/L), while tube B received 1 mL of boric acid–sodium acetate solution (30 g/L boric acid in 250 g/L sodium acetate). Both tubes were incubated in the dark for 20 min. Then, 1 mL of o-phenylenediamine (0.2 g/L) was added to each tube, followed by an additional 40 min incubation in the dark. The fluorescence intensity was measured using a multimode reader (BioTek Synergy LX, BioTek Instruments, Inc., Winooski, VT, USA) with excitation and emission wavelengths set at 355 nm and 425 nm, respectively. The results were expressed as mg 100 g^−1^ on the basis of fresh weight. All measurements were performed in triplicate.

### 2.7. Determination of O*_2_*^•−^ and H*_2_*O*_2_* Content

Levels of O_2_^•−^ and H_2_O_2_ concentrations in Chinese flowering cabbage stem were quantified utilizing commercial diagnostic kits (Suzhou Keming Biotechnology Co., Ltd., Suzhou, China) as per the manufacturer’s guidelines. Briefly, 0.1 g of frozen tissue was homogenized in an ice-chilled environment with 1 mL of the specialized extraction buffer in a 2 mL tube. Following a 20-min centrifugation at 12,000× *g* and 4 °C, the supernatant was harvested for subsequent detection. H_2_O_2_ levels were assessed through its reaction with titanium sulfate, yielding a yellow complex measurable at 415 nm. Data for H_2_O_2_ were expressed as µmol g^−1^ FW. O_2_^•−^ concentrations were determined by the reaction with hydroxylamine hydrochloride, resulting in a red derivative with an absorbance peak at 530 nm, with the results reported as nmol g^−1^ FW.

### 2.8. Assay of MDA Content

Malondialdehyde (MDA) levels were evaluated following the protocol of Tan et al. (2020) [27] with minor refinements. Briefly, 0.2 g of Chinese flowering cabbage stem powder was homogenized in 1 mL of 6.7 g/L thiobarbituric acid (TBA) solution for extraction. The mixture was centrifuged at 10,000× *g* for 20 min at 4 °C. The resulting supernatant was mixed with 0.67% TBA solution, vortexed thoroughly, and then heated in a boiling water bath for 20 min. After cooling, the mixture was centrifuged again, and the absorbance of the supernatant was measured at 450, 532, and 600 nm. The MDA concentration was calculated and expressed as nmol g^−1^ on a fresh weight basis.

### 2.9. Measurement of ROS-Related Enzyme Activities

POD activity was quantified by modifying the protocol established by Wang et al. (2020) [2] with minor modifications. Briefly, 0.1 g of frozen Chinese flowering cabbage stalk was homogenized in 1 mL of an extraction medium (comprising 1% PEG, 4% PVPP, and 1% Triton X-100) on ice. Centrifugation of the homogenate was performed at 12,000× *g* for 30 min at 4 °C. To start the enzymatic reaction, a 100 µL aliquot of the extract was blended with 3.0 mL of 25 mmol/L guaiacol and 200 µL of 0.5 mol/L H_2_O_2_. Absorbance at 420 nm was tracked for 3 min, and the results were reported as U g^−1^ on a fresh weight (FW) basis.

SOD activity was determined according to the method of Ma et al. (2018) [28] based on the inhibition of the photochemical reduction of nitro blue tetrazolium (NBT) by O_2_^•−^. Take 100 μL of crude enzyme extract and add it to a reaction mixture consisting of 130 mM methionine (Met), 750 µM nitroblue tetrazolium (NBT), 100 µM EDTA-Na_2_, and 20 µM riboflavin. Mix well, then perform the reaction under light, and measure at 560 nm. One unit (U) of SOD activity was indicated as the amount of enzyme causing 50% inhibition of NBT photoreduction.

CAT activity was assessed based on the procedure described by Nomura et al. (2017) [29]. Frozen stalk samples (0.1 g) were homogenized in an ice bath with 1 mL of extraction buffer containing 5 mmol/L DTT and 5% PVP. Following centrifugation at 12,000× *g* for 30 min at 4 °C, a 100 µL volume of the supernatant was combined with 2.9 mL of 20 mmol/L H_2_O_2_. The reduction in absorbance at 240 nm was monitored, and activity was expressed as U g^−1^ FW.

### 2.10. Real-Time Quantitative RT-PCR Analysis

RT-qPCR was performed according to the protocols described by Wang et al. (2023) [5]. Total RNA was extracted from stem tissues using a Quick RNA Isolation Kit (Tiangen, Beijing, China). The purity and integrity of the extracted RNA were verified using a NanoDrop spectrophotometer (Thermo Fisher Scientific, Waltham, MA, USA). Subsequently, 1 µg of total RNA was reverse-transcribed into first-strand cDNA in a 10 µL reaction volume using a PrimeScript™ RT Master Mix (Takara, Kusatsu, Japan) following the manufacturer’s instructions.

The RT-qPCR assays were conducted on a CFX Connect Real-Time PCR System (Bio-Rad, Hercules, CA, USA) using a SYBR^®^ Premix Ex Taq™ kit (Takara, Kusatsu, Japan). The RT-qPCR assays were performed to quantify the relative expression levels of genes involved in ROS metabolism, including antioxidant-related enzymes (*BrSOD*, *BrCAT*, and *BrPOD*) and NADPH oxidase-related genes (*BrRbohC* and *BrRbohD*). The sequences of these genes were obtained from previous studies on *Brassica rapa*. All primers used in this study (Supplementary Table S1) were synthesized by Sangon Biotech Co., Ltd. (Guangzhou, China). The Actin gene was used as an internal control to normalize the expression data. The relative expression levels of target genes were calculated using the $2^{-{\Delta \Delta C}_{T}}$ method [30]. The primers used in this study are listed in Supplementary Table S1 [5,31].

### 2.11. Statistical Analysis

The experiment was conducted using a completely randomized design (CRD) with a 2 × 2 factorial arrangement, consisting of two packaging types (PE and MAP) and two electrostatic field levels (0 and 1 kV). Statistical analyses were performed using SPSS 24.0, where significant differences between treatments were determined by one-way analysis of variance (ANOVA) followed by a post hoc test at a significance level of *p* < 0.05 and high significance at *p* < 0.01. For multivariate evaluation, principal component analysis (PCA) and correlation heatmaps were generated, principal components with eigenvalues > 1 were extracted to calculate variance-weighted comprehensive scores (SPSSAU project 26.0, 2026) [32]. All graphical representations and mappings were prepared using GraphPad Prism 10.2 and Origin 2024b.

## 3. Results

### 3.1. Effects of LVEF+MAP Treatment on Color, Chlorophyll Content, and Weight Loss of Chinese Flowering Cabbage During Storage

**Figure 1:**
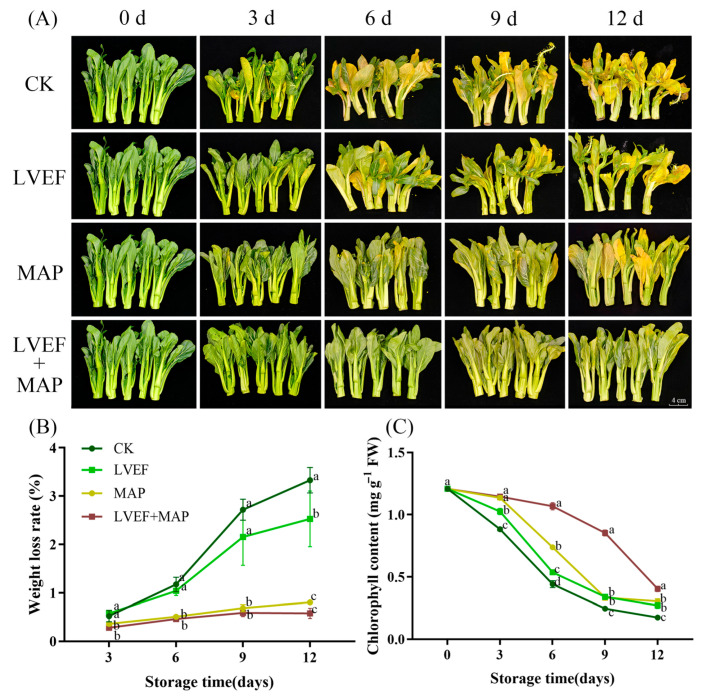
The effects of LVEF+MAP treatment on the appearance (**A**), weight loss (**B**), and chlorophyll content (**C**) of Chinese flowering cabbage during storage. Different lowercase letters at the same storage time point indicate significant differences among the four treatments (*p* < 0.05). Scale bar = 2 cm.

Leaf yellowing indicates sensory quality and commercial value in Chinese flowering cabbage, with chlorophyll content determining greenness [33]. As shown in Figure 1A, the leaves of Chinese flowering cabbage in the CK, LVEF, and MAP groups exhibited varying degrees of yellowing from day 3 onward, whereas obvious yellowing in the LVEF+MAP group was not observed until day 9, indicating that the combined treatment effectively delayed leaf degreening. By day 12 of storage, the leaves in the CK group were almost completely yellow and showed severe stalk bending. In contrast, stalk bending was alleviated in the LVEF group, while no obvious severe bending was observed in either the MAP or LVEF+MAP group. In addition, LVEF+MAP treatment was more effective than MAP alone in maintaining leaf greenness. These results indicated that the combined application of the LVEF and MAP was more effective in preserving the sensory quality of postharvest Chinese flowering cabbage.

In agreement with the above visual observations, chlorophyll content declined in all groups during storage, although the extent of decline differed significantly among treatments (Figure 1C). By day 6, the CK group exhibited the greatest reduction in chlorophyll content, showing a 63.43% decrease relative to the initial level. The corresponding decreases in the LVEF, MAP, and LVEF+MAP groups were 55.51%, 38.92%, and 11.54%, respectively, with significant differences observed among the four groups (*p* < 0.05). From day 6 onward, the chlorophyll content in the LVEF+MAP group remained significantly higher than that in the other three groups, indicating that the combined treatment was the most effective in delaying chlorophyll degradation during storage.

Weight loss during postharvest storage of fruit and vegetables is mainly attributed to water loss and the consumption of organic substrates resulting from transpiration and respiration [34]. As shown in Figure 1B, the weight loss rates in the CK and LVEF groups increased rapidly throughout storage, whereas that in the MAP and LVEF+MAP groups increased more slowly. At the end of storage, the weight loss rates in the CK, LVEF, MAP, and LVEF+MAP groups reached 3.33%, 2.53%, 0.80%, and 0.58%, respectively. Notably, both the MAP and LVEF+MAP groups showed significantly lower weight loss rates than the CK group throughout storage (*p* < 0.05), suggesting that MAP was primarily responsible for limiting postharvest weight loss.

### 3.2. The Effects of LVEF+MAP Treatment on O*_2_* and CO*_2_* Concentrations Inside the Packages of Chinese Flowering Cabbage During Storage

**Figure 2:**
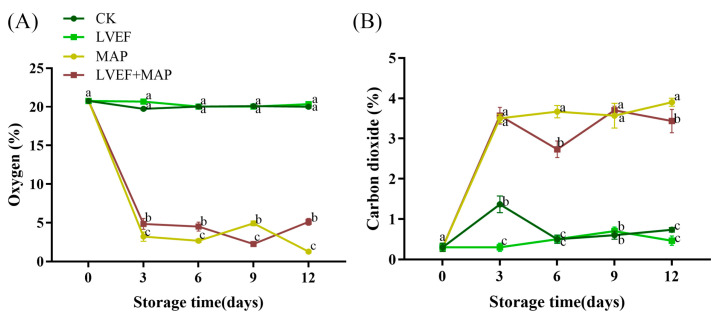
The effects of LVEF+MAP treatment on O_2_ concentration (**A**) and CO_2_ concentration (**B**) inside the packages of Chinese flowering cabbage during storage. Different lowercase letters at the same storage time point indicate significant differences among the four treatments (*p* < 0.05).

Changes in the gas composition inside the packages can regulate the respiratory metabolism of postharvest vegetables, thereby affecting their senescence process and quality maintenance [35]. As shown in Figure 2A, in the perforated PE bags used for the CK and LVEF groups, the O_2_ concentration remained relatively stable during storage, indicating that these bags did not establish a modified atmosphere. In contrast, the O_2_ concentration in the MAP and LVEF+MAP groups packaged with MP30 decreased rapidly in the early period during storage. By day 3, the O_2_ concentrations inside the packages of the CK, LVEF, MAP, and LVEF+MAP groups decreased from the initial 20.77% to 19.73%, 20.66%, 3.22%, and 4.85%, respectively. By day 12, the corresponding values were 20.00%, 20.33%, 1.28%, and 5.11%, respectively. The difference between the CK and LVEF groups was small, whereas the LVEF+MAP group maintained a significantly higher O_2_^•−^ concentration than the MAP group (*p* < 0.05).

Similarly, as shown in Figure 2B, the CO_2_ concentrations changed only slightly in the perforated PE bags of the CK and LVEF groups, but increased markedly in the MAP and LVEF+MAP groups. By day 12, the CO_2_ concentrations in the CK, LVEF, MAP, and LVEF+MAP groups increased from the initial 0.30% to 0.73%, 0.47%, 3.90%, and 3.43%, respectively. The MAP group showed a significantly higher CO_2_ concentration than the LVEF+MAP group (*p* < 0.05). Consequently, the LVEF+MAP group exhibited a higher O_2_ concentration and lower CO_2_ accumulation than the MAP group.

### 3.3. Effects of LVEF+MAP Treatment on Stem Hollowing, Soluble Protein Content, and Vitamin C Content of Chinese Flowering Cabbage During Storage

**Figure 3:**
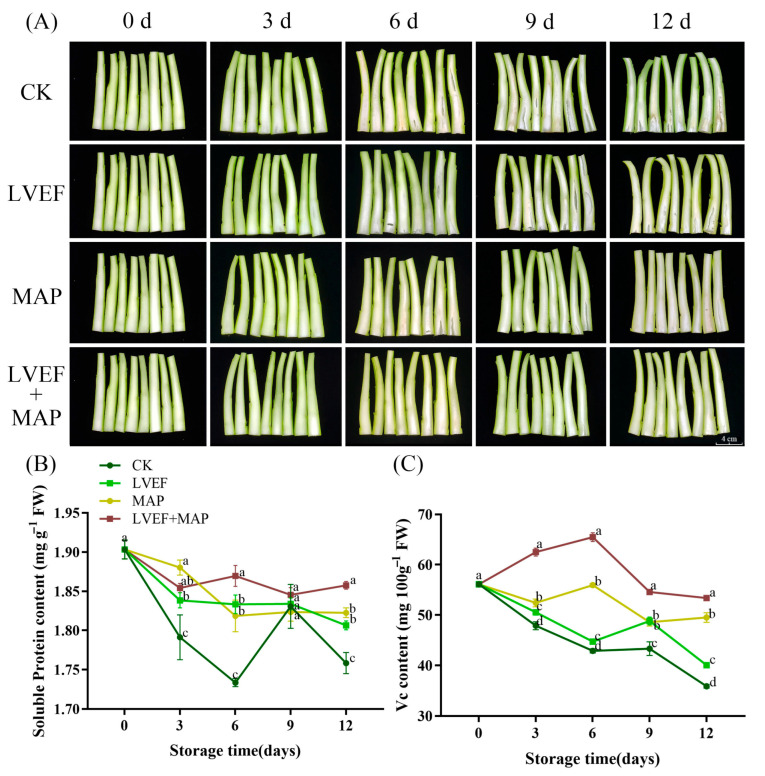
The effects of LVEF+MAP treatment on stem hollowing (**A**), soluble protein content (**B**), and Vitamin C content (**C**) of Chinese flowering cabbage during storage. Different lowercase letters at the same storage time point indicate significant differences among the four treatments (*p* < 0.05). Scale bar = 2 cm.

The stem is the main edible part of Chinese flowering cabbage, and its pith phenotype is shown in Figure 3A. Stem hollowing in the CK group was observed as early as day 3 of storage. By day 12, all stems in the CK group showed obvious hollowing, whereas the LVEF group exhibited less sever hollowing. In the MAP group, hollowing was first observed on day 6, while no obvious hollowing was observed in the LVEF+MAP group even at the end of storage.

As shown in Figure 3B, the soluble protein content in the stems of Chinese flowering cabbage exhibited an overall downward trend in all treatments during storage. By day 6, the soluble protein contents in the CK, LVEF, MAP, and LVEF+MAP groups decreased by 9%, 4%, 4%, and 2%, respectively. By day 12, the soluble protein content in the LVEF and MAP groups was higher than that in the CK group (*p* < 0.05), while that in the LVEF+MAP group was higher than that in the MAP group (*p* < 0.05). These results suggest that LVEF treatment apparently inhibits the decline in soluble protein content and therefore maintains postharvest quality. Moreover, the combination of the LVEF and MAP showed a greater effect than either treatment alone, indicating a synergistic role in preserving soluble protein in Chinese flowering cabbage during storage.

As illustrated in Figure 3C, the Vitamin C content of the stem in the LVEF+MAP group increased slightly during the early stage of storage, peaking at 65.49 mg/100 g on day 6, whereas the CK, LVEF, and MAP groups showed an overall decreasing trend. By day 12, the Vitamin C contents in the CK, LVEF, MAP, and LVEF+MAP groups decreased by 36.04%, 28.66%, 11.78%, and 4.89%, respectively, from the initial content of 56.12 mg/100 g. Throughout the storage period, the LVEF+MAP group consistently maintained the highest Vitamin C content, significantly exceeding all other groups (*p* < 0.05). Furthermore, the LVEF and MAP groups retained a significantly higher Vitamin C content than the CK group (*p* < 0.05). These results indicate that both MAP and LVEF treatment effectively delayed Vitamin C degradation in Chinese flowering cabbage during postharvest storage, and their combination further enhanced this preservation effect.

### 3.4. Effects of LVEF+MAP Treatment on O*_2_*^•−^ Content, H*_2_*O*_2_* Content, MDA Content, and Antioxidant Enzyme Activities in Stems of Chinese Flowering Cabbage During Storage

**Figure 4:**
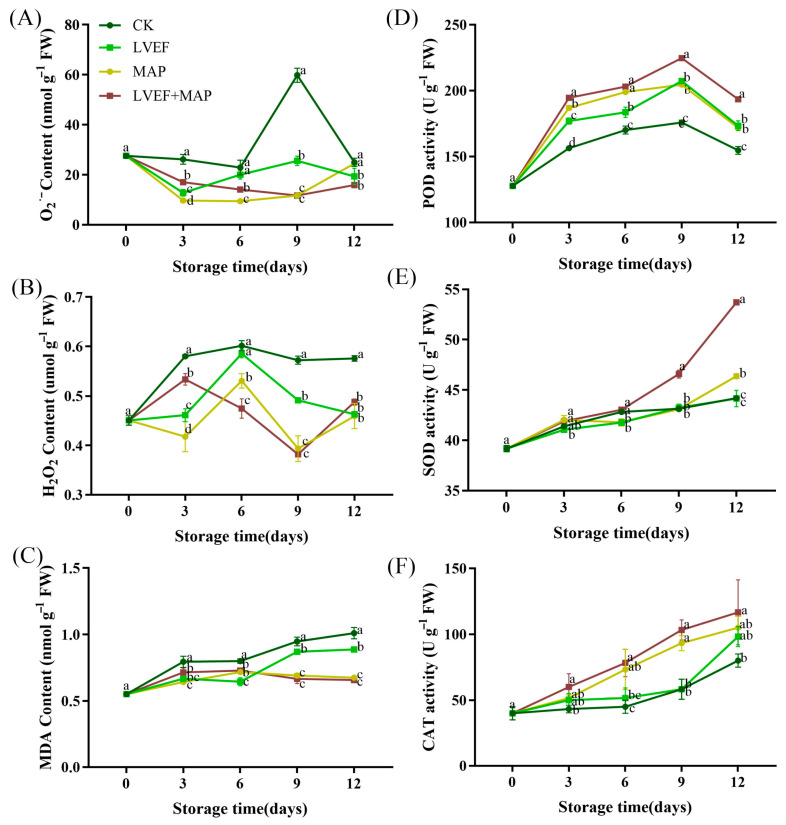
The effects of LVEF+MAP treatment on the O_2_^•−^ generation rate (**A**), H_2_O_2_ content (**B**), MDA content (**C**), POD activity (**D**), SOD activity (**E**), and CAT activity (**F**) of Chinese flowering cabbage during storage. Different lowercase letters at the same storage time point indicate significant differences among the four treatments (*p* < 0.05).

Postharvest senescence is often accompanied by excessive accumulation of ROS, such as O_2_^•−^ and H_2_O_2_ [36]. As shown in Figure 4A, the O_2_^•−^ content in the stem of the CK group remained higher than that in all other treatment groups throughout storage. The CK group peaked at 59.80 nmol/g on day 9, after which it decreased by 58.21% by day 12, a decline that may be attributed to extensive cell death and reduced metabolic activity during the late storage period. By day 12, the O_2_^•−^ contents in the CK, LVEF, MAP, and LVEF+MAP groups were 24.99, 19.34, 24.50, and 15.87 nmol/g, respectively. Compared with the CK group, the LVEF, MAP, and LVEF+MAP groups maintained lower O_2_^•−^ levels, with the LVEF+MAP group being significantly lower than the other two treatment groups *(p* < 0.05). Overall, the H_2_O_2_ content in the CK group remained higher than that in the LVEF, MAP, and LVEF+MAP groups throughout the storage period. On day 6, the H_2_O_2_ content in the MAP group was 11.82% higher than that in the LVEF+MAP group, while on day 9, the CK group exhibited an H_2_O_2_ content 16.49% higher than that of the LVEF group (Figure 4B). These results indicate that LVEF treatment, particularly when combined with MAP, effectively suppressed the accumulation of O_2_^•−^ and H_2_O_2_, thereby maintaining lower ROS levels and delaying stem senescence in Chinese flowering cabbage. MDA is commonly used as an indicator of oxidative damage to cell membranes [37]. As shown in Figure 4C, the CK group consistently exhibited the highest MDA content throughout storage, and the differences from the other treatment groups became significant from day 3 onward (*p* < 0.05). The MAP and LVEF+MAP groups showed relatively small differences in MDA content during storage, suggesting that these two treatments may exert similar inhibitory effects on MDA accumulation, without an obvious additive effect under combined treatment.

To elucidate the relationship between antioxidant enzymes and postharvest quality, we investigated their activities in Chinese flowering cabbage. As shown in Figure 4D, the POD activity in all treatment groups increased first and then decreased during storage, reaching a maximum on day 9. At this time, the POD activities in the CK, LVEF, MAP, and LVEF+MAP groups were 175.80, 207.37, 204.60, and 224.73 U g^−1^ FW, respectively. Moreover, POD activity was higher in both the LVEF and MAP groups than in the CK group, and was further increased in the LVEF+MAP group (*p* < 0.05). As illustrated in Figure 4E, SOD activity in Chinese flowering cabbage generally increased during storage. By day 12, the SOD activities in the CK, LVEF, MAP, and LVEF+MAP groups increased by 12.81%, 12.68%, 18.35%, and 37.08%, respectively, from the initial value of 39.18 U g^−1^FW. Notably, the LVEF+MAP samples showed the highest SOD activity throughout storage and was higher than the other treatment groups (*p* < 0.05). Similarly, CAT activity increased during storage (Figure 4F). By day 12, CAT activity was 22.91% and 31.25% higher in the LVEF and MAP groups, respectively, than in the CK group. In addition, CAT activity in the LVEF+MAP group was 11.11% higher than that in the MAP group and 18.65% higher than that in the LVEF group. These results indicate that LVEF treatment was beneficial for maintaining higher CAT activity, and this effect became more pronounced when combined with MAP.

### 3.5. The Effects of LVEF+MAP Treatment on the Expression of BrRboh Genes and Antioxidant Enzyme-Related Genes in the Stems of Chinese Flowering Cabbage During Storage

**Figure 5:**
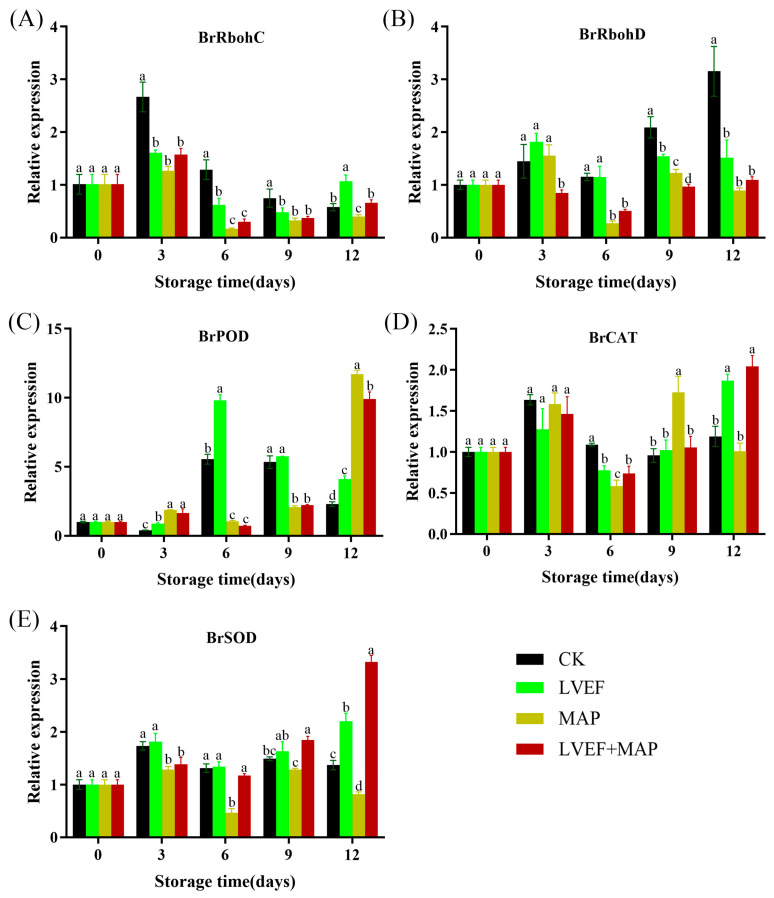
The effects of LVEF+MAP treatment on the relative expression levels of ROS metabolism-related genes, including *BrRbohC* (**A**)*, BrRbohD* (**B**), *BrPOD* (**C**), *BrCAT* (**D**) and *BrSOD* (**E**), in the stem of Chinese flowering cabbage during storage. Different lowercase letters at the same storage time point indicate significant differences among the four treatments (*p* < 0.05).

ROS homeostasis, which involves a dynamic balance between ROS generation and scavenging, plays a critical role in regulating the postharvest quality of Chinese flowering cabbage [31]. The rapid accumulation of ROS in postharvest stems was primarily mediated by the activation of respiratory burst oxidase homologs (*RBOH*s), which serves as a major enzymatic source for ROS generation during tissue deterioration. During senescence of Chinese flowering cabbage, *BrRbohC* expression initially increased rapidly, peaking on day 3 (Figure 5A). The expression level in the CK group was the highest among all groups, with no significant differences among the other three groups (*p* > 0.01), after which it declined. By day 9, *BrRbohC* expression in the CK, LVEF, MAP, and LVEF+MAP groups decreased by 71.91%, 70.19%, 73.81%, and 76.43%, respectively. In contrast, *BrRbohD* expression (Figure 5B) exhibited a marked increasing trend in the CK group and a moderate increase in the LVEF group, whereas it remained relatively stable or was suppressed in the MAP and LVEF+MAP groups throughout the storage period. By day 9, the expression levels in the CK, LVEF, MAP, and LVEF+MAP groups were 3.15, 1.54, 0.90, and 1.09 times that at day 0, respectively. These changes indicate that both LVEF and MAP treatments alone, as well as their combination, suppressed the increase in *BrRbohD* expression, with the combined treatment exerting a stronger inhibitory effect. These results suggest that LVEF+MAP treatment may reduce NADPH oxidase activity by suppressing the expression of *RBOH*-related genes, thereby decreasing ROS generation and delaying postharvest senescence in Chinese flowering cabbage.

The enzymatic antioxidant defense system, primarily comprising SOD, CAT, and POD, plays pivotal role in scavenging ROS radicals and maintaining cellular redox homeostasis [38,39]. As shown in Figure 5C, *BrPOD* expression in both the CK and LVEF groups exhibited a trend of first increasing and then decreasing, peaking on day 6 with expression levels of 5.55 and 9.81, respectively. In contrast, *BrPOD* expression in the MAP and LVEF+MAP groups reached the highest level on day 12, representing 10.72-fold and 8.90-fold increases relative to day 0, respectively. During the late stage of storage, *BrCAT* expression in the LVEF and LVEF+MAP groups was higher than that in CK and MAP groups (Figure 5D). By day 12, the expression levels of *BrCAT* in the CK, LVEF, MAP, and LVEF+MAP groups were 1.19, 1.87, 1.01, and 2.04, respectively. Throughout the storage period, *BrSOD* expression showed an upward trend (Figure 5E), and in the LVEF and LVEF+MAP groups, it remained generally higher than in the CK and MAP groups. By day 12, the expression levels in the four groups were 1.37, 2.20, 0.82, and 3.33, respectively, with the levels in the LVEF+MAP group being significantly higher compared to the other treatment groups *(p* < 0.05). Overall, both LVEF and MAP treatments induced the expression of *BrPOD*, *BrCAT*, and *BrSOD*, whereas during the late storage stage, the LVEF+MAP treatment showed the more pronounced effect in maintaining *BrCAT* and *BrSOD* expression, which was generally consistent with the trends observed in enzyme activities.

### 3.6. Comprehensive Evaluation of the Effects of LVEF+MAP Treatment on the Postharvest Quality of Chinese Flowering Cabbage Stems

**Figure 6:**
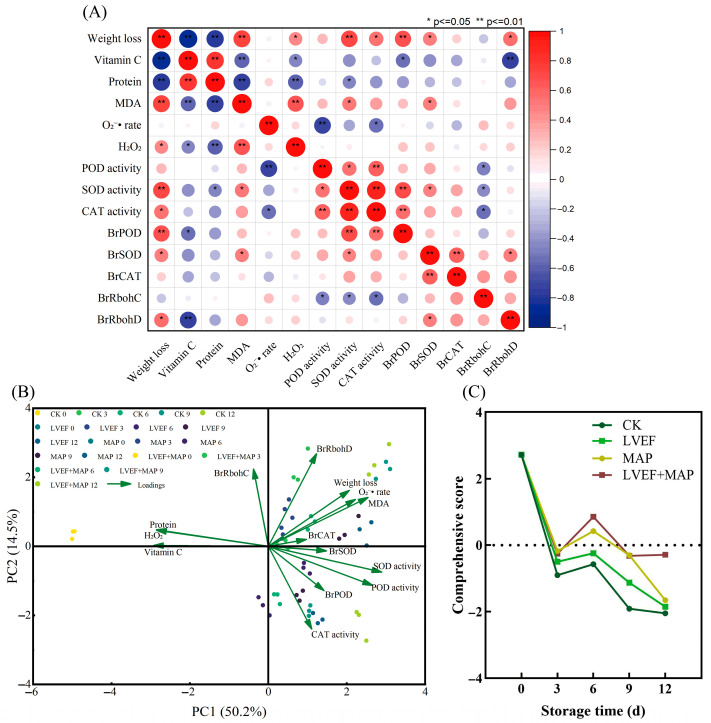
Correlation analysis (**A**), principal component analysis (**B**), and comprehensive evaluation of quality-related indices (**C**) of Chinese flowering cabbage under different treatments during storage. In panel A, red indicates a positive correlation, blue indicates a negative correlation, and circle size represents the strength of the correlation. * and ** indicate significance at *p* ≤ 0.05 and *p* ≤ 0.01, respectively.

As shown in Figure 6A, weight loss was significantly positively correlated with MDA content, whereas it was significantly negatively correlated with Vitamin C and protein contents, indicating that increased water loss was closely associated with membrane lipid peroxidation and nutritional deterioration during storage. MDA was negatively correlated with Vitamin C and protein content, further suggesting that oxidative damage accelerated quality loss in Chinese flowering cabbage. In addition, the O_2_^•−^ generation rate and H_2_O_2_ content showed positive correlations with weight loss and MDA, indicating that ROS accumulation played an important role in postharvest senescence. POD, SOD, and CAT activities were positively correlated with each other and generally positively associated with the expression levels of their corresponding genes, suggesting a coordinated antioxidant defense response.

The PCA results (Figure 6B) show that PC1 and PC2 explained 50.2% and 14.5% of the total variance, respectively. Chinese flowering cabbage at day 0 was mainly distributed on the negative side of PC1 and closely associated with Vitamin C and protein, reflecting better initial quality. With prolonged storage, samples gradually shifted toward the positive side of PC1, where weight loss, MDA, and the O_2_^•−^ generation rate were located, indicating progressive quality deterioration and oxidative damage. The separation among treatments suggests that LVEF and MAP treatments, particularly their combination, effectively modulated postharvest physiological changes. As shown in Figure 6C, the comprehensive score of all groups decreased during storage, but the decline was markedly alleviated by LVEF and MAP treatments. Among all treatments, the LVEF+MAP group maintained the highest comprehensive score during the middle and late stages of storage, indicating that the combined treatment had the best effect in preserving the postharvest quality of Chinese flowering cabbage.

## 4. Discussion

Postharvest senescence of Chinese flowering cabbage is a complex process involving water loss, respiratory metabolism, nutrient depletion, oxidative damage, and the progressive collapse of cellular homeostasis [5,8,31]. In the present study, the combined treatment of a low-voltage electrostatic field (LVEF) and modified atmosphere packaging (MAP) effectively delayed quality deterioration during storage [22,23]. Since harvested vegetables remain physiologically active, continuous respiration and metabolic activity accelerate substrate consumption and senescence progression [39]. Therefore, regulation of the gaseous environment inside the package, particularly O_2_ and CO_2_ concentrations, is an important way to maintain postharvest quality [35]. Low O_2_ and moderately high CO_2_ conditions generally reduce the respiration rate of vegetables and decrease substrate consumption [40]. Our previous study demonstrated that the MAP film could effectively delay senescence in Chinese flowering cabbage [6]. In the present study, both MAP and the LVEF created a favorable low-O_2_/high-CO_2_ microenvironment (Figure 2A,B), which likely provided the physiological basis for delayed senescence. This effect may be partly attributed to the relatively low gas permeability of the MP30 film, which restricted gas exchange between the package interior and the external atmosphere [41]. At the same time, the distinct gas composition observed in the LVEF+MAP treatment suggests that LVEF may have further influenced the in-package atmosphere by inhibiting respiratory activity, thereby reducing O_2_ consumption and CO_2_ production. The LVEF+MAP group maintained relatively higher O_2_ levels and lower CO_2_ accumulation than the MAP group during storage (Figure 2A,B). The comparable inhibition of weight loss in the two MAP treatments indicates that MAP was the principal factor limiting postharvest water loss, most likely through the maintenance of a stable package microenvironment that reduced transpiration. In contrast, the additional contribution of the LVEF appears to be more closely associated with the preservation of chlorophyll and the maintenance of tissue physiological integrity, thereby delaying leaf yellowing and preserving visual quality (Figure 1). Collectively, these findings suggest that the senescence-delaying effect of LVEF+MAP resulted from the coordinated action of external atmospheric regulation by MAP and internal physiological modulation by the LVEF. From these results, the role of the LVEF may not simply add to that of MAP, but rather modulate the metabolism of the produce under an already established modified atmosphere. This effect is similar to those of many reported combined preservation technologies, which are typically founded on appropriate packaging strategies. For example, a Light-Emitting Diode (LED) combined with MAP delays quality deterioration in Chinese flowering cabbage and okra [42,43]. A high-voltage electrostatic field combined with MAP could effectively extend the shelf life of pak choi and avoid off-flavors [44].

Beyond maintaining leaf appearance and regulating in-package atmosphere, preserving stem quality is particularly important for Chinese flowering cabbage, as the stem is the main edible organ and determines consumer acceptability [2,41]. Stem hollowing develops subtly, often before visible leaf senescence, and is characterized by central pith and tissue loosening [5]. In this study, LVEF and MAP treatments, especially their combination, markedly reduced stem hollowing (Figure 3A), indicating effective alleviation of structural deterioration during storage. This may be associated with improved water status and suppression of senescence-related cell wall disassembly, thereby delaying pith loosening and cavity development. In addition to structural integrity, the nutritional quality of the flowering stem is another important determinant of postharvest value. As an important indicator of postharvest quality and metabolic status, soluble protein decreases significantly in Chinese flowering cabbage after the onset of senescence [45]. The higher soluble protein content under LVEF treatment (Figure 3B) suggests that the LVEF delayed protein degradation and helped maintain metabolic activity in stem tissues. Similar effects have been reported in sweet corn, where an LVEF reduced postharvest sugar metabolism and enhanced stress resistance [46]. Notably, the combined treatment showed a greater preservative effect than either treatment alone (Figure 3B), suggesting a synergistic role of the LVEF and MAP in retarding nutrient loss. A similar pattern was observed for Vitamin C, which is highly susceptible to oxidative degradation during storage. MAP and LVEF treatments improved Vitamin C retention (Figure 3C), particularly when combined, indicating reduced oxidative consumption and better preservation of stem nutritional quality. Specifically, the initial increase in Vitamin C and soluble protein levels observed in the LVEF+MAP group suggests that the treatment may act as a mild abiotic stress. This stimulus likely activated the endogenous defense signaling pathways, promoting the synthesis or regeneration of these components to counteract postharvest oxidative stress, while the MAP-stabilized microenvironment further supported this metabolic response. As the stem is rich in Vitamin C, this compound contributes not only to nutritional value but also to ROS scavenging and redox homeostasis, both of which are closely associated with senescence progression [47]. Taken together, these findings indicate that the beneficial effect of LVEF+MAP on Chinese flowering cabbage extended beyond delaying external senescence, encompassing the maintenance of stem structure and nutritional status, thereby better preserving edible quality during storage.

**Figure 7:**
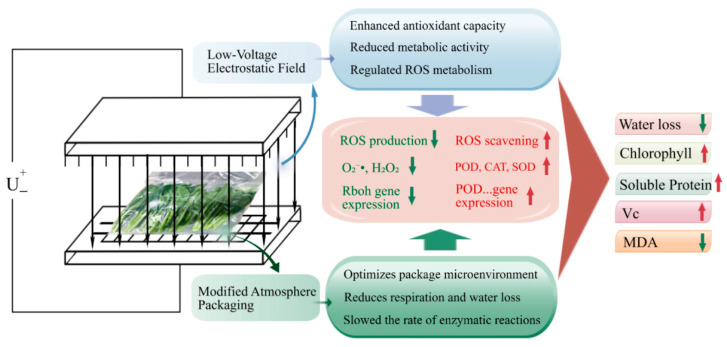
Proposed mechanism by which LVEF+MAP treatment maintains postharvest quality of Chinese flowering cabbage during storage. The red upward arrows and green downward arrows indicate the increase and decrease of the corresponding indices, respectively.

Moderate ROS levels act as signaling molecules in normal ripening and senescence, but excessive ROS promote quality deterioration [36]. When ROS overaccumulation overwhelms the antioxidant system, oxidative stress occurs, leading to membrane lipid peroxidation, protein oxidation, enzyme inactivation, and accelerated tissue deterioration [48]. Our correlation analysis further revealed that MDA and ROS accumulation (O_2_^•−^ and H_2_O_2_) were negatively correlated with Vitamin C and protein contents, suggesting that oxidative stress is a driving force behind nutritional degradation (Figure 6A). In the present study, control stem samples exhibited higher O_2_^•−^ generation and H_2_O_2_ content during storage, accompanied by the marked increase in MDA, a key indicator of membrane lipid peroxidation. In contrast, LVEF, MAP, and LVEF+MAP treatments significantly suppressed ROS accumulation and maintained lower MDA levels (Figure 4A–C), indicating effective alleviation of oxidative injury and preservation of membrane integrity. This protective effect was closely associated with enhanced activities of SOD, POD, and CAT (Figure 4D–F). SOD mainly functions to catalyze the dismutation of O_2_^•−^ into H_2_O_2_, while CAT and POD further decompose H_2_O_2_ into water and oxygen [49]. The higher expression levels of genes such as *BrSOD*, *BrCAT*, and *BrPOD* were consistent with the increased activities of their corresponding enzymes (Figure 5C–E), indicating that the antioxidant response was regulated, at least partially, at the transcriptional level. The coordinated upregulation of these enzymes and the corresponding gene expression suggests that LVEF+MAP promoted a more efficient ROS-scavenging network, enabling timely removal of excess ROS during storage. Previous studies have reported that an LVEF enhances the antioxidant system, reduces oxidative damage, and improves the quality of strawberries and bell peppers [11,16]. Similarly, MAP preserves the metabolic balance of ROS by modulating the respiration of fruits and vegetables [18]. In addition to scavenging, the expression patterns of *BrRbohC* and *BrRbohD* (Figure 5A,B), which are related to ROS generation through plasma membrane NADPH oxidases (RBOHs), provide further insight into ROS homeostasis [50]. Correspondingly, *BrRbohC* and *BrRbohD* showed overall higher expression levels in the CK group, particularly BrRbohD, which exhibited a more pronounced increase during the late storage stage, while LVEF, MAP, and LVEF+MAP treatments significantly suppressed their expression; in particular, LVEF+MAP treatment showed the most pronounced inhibitory effect (Figure 5A,B). The members of the RBOH family play important roles in ROS production and plant responses to environmental stress, and their high expression generally indicates that tissues are subjected to stronger stress stimulation accompanied by oxidative burst [39]. Postharvest preservation is not achieved by completely eliminating ROS, but rather by maintaining them at a controlled level where they function as signaling molecules without causing detrimental oxidative damage [36]. Taken together, these findings suggest that the LVEF combined with MAP may act as a mild abiotic stimulus that activates the endogenous defense system of Chinese flowering cabbage MAP establishes a favorable external atmosphere and moisture-retaining condition, whereas the LVEF may further induce internal defense signaling and strengthen antioxidant capacity (Figure 7). This integrated approach leads to a complete regulatory chain from gene expression to enzyme activity, and from metabolite preservation to phenotype maintenance.

## 5. Conclusions

This study comprehensively investigated the synergistic effects of a low-voltage electrostatic field (LVEF) and modified atmosphere packaging (MAP) on the postharvest quality and senescence of Chinese flowering cabbage. Our findings demonstrate that the combined LVEF+MAP treatment significantly outperformed individual applications by effectively mitigating weight loss, delaying stem hollowing, and preserving critical nutritional components such as soluble protein and Vitamin C.

LVEF+MAP treatment synergistically activated the endogenous antioxidant defense system of Chinese flowering cabbage. This was evidenced by the remarkable suppression of ROS accumulation (O_2_^•−^ and H_2_O_2_) and membrane lipid peroxidation (MDA), coupled with enhanced activities of key antioxidant enzymes (SOD, POD, and CAT). Crucially, these physiological improvements were underpinned by molecular regulation, as we observed coordinated upregulation of antioxidant enzyme-related genes (*BrSOD*, *BrCAT*, *BrPOD*) and modulated expression of ROS-generating genes (*BrRbohC*, *BrRbohD*). This suggests that LVEF+MAP treatment not only provides a protective external environment but also actively rebalances the plant’s internal ROS homeostasis at the transcriptional level, thereby delaying senescence at its fundamental molecular roots. The combined LVEF+MAP treatment extended the commercially acceptable shelf-life of Chinese flowering cabbage to 12 days. Our results highlight LVEF+MAP treatment as a promising, environmentally friendly, and highly effective strategy for extending the shelf life and maintaining the quality of Chinese flowering cabbage, offering valuable insights for the postharvest preservation of similar specialty vegetables.

## Data Availability

The original contributions presented in this study are included in the article/Supplementary Material. Further inquiries can be directed to the corresponding authors.

## Supplementary Materials

The following supporting information can be downloaded at: https://www.mdpi.com/article/10.3390/foods15101674/s1, Table S1. List of primers used in quantitative real-time PCR, Figure S1. Preliminary observation of the effects of different LVEF intensities (0.5, 1, and 1.5 kV) combined with MAP on the visual quality of Chinese flowering cabbage during storage (0–6 days), Figure S2. Visual representation of Chinese flowering cabbage samples in packaging bags under different treatments at 12 d of storage (CK, LVEF, MAP, and LVEF+MAP).
