# Supplementary material for: Effects of Low-Voltage Electrostatic Field Combined with Modified Atmosphere Packaging on Postharvest Quality and Senescence of Chinese Flowering Cabbage
Source: Foods. 2026 May 11;15(10):1674. doi: 10.3390/foods15101674 (PMC13205157; doi:10.3390/foods15101674)
Supplement: Supplementary file 1 [file foods-15-01674-s001.zip › foods-4278651-supplementary.pdf]

Table S1. List of primers used in quantitative real-time PCR

Gene Name	Primer sequence (5'–3') (Sense/Anti-Sense)
BrPOD	CCGCTTTCTCTTGGCTCCGTAAC
	GCTCCTGTCGTGTTCTTTCTCTCC
BrCAT	TCAAGTTTTACACCCGAGAG
	CATCATCAAACATCCAGCAC
BrSOD	GCACCCGAGGATGCTAATCG
	TACCACAAGCAACACGGCCT
BrRbohC	GGATGGTCTGCCGTGGAGAA
	CGTCTCGCCAACGCATCAAA
BrRbohD	GGAGAGGCTGATCCGTGCTT
	GTCTCCTGGTGCGGATGTGA
BrActin	GGAGCTGAGAGATTCCGTTG
	GAACCACCACTGAGGACGAT

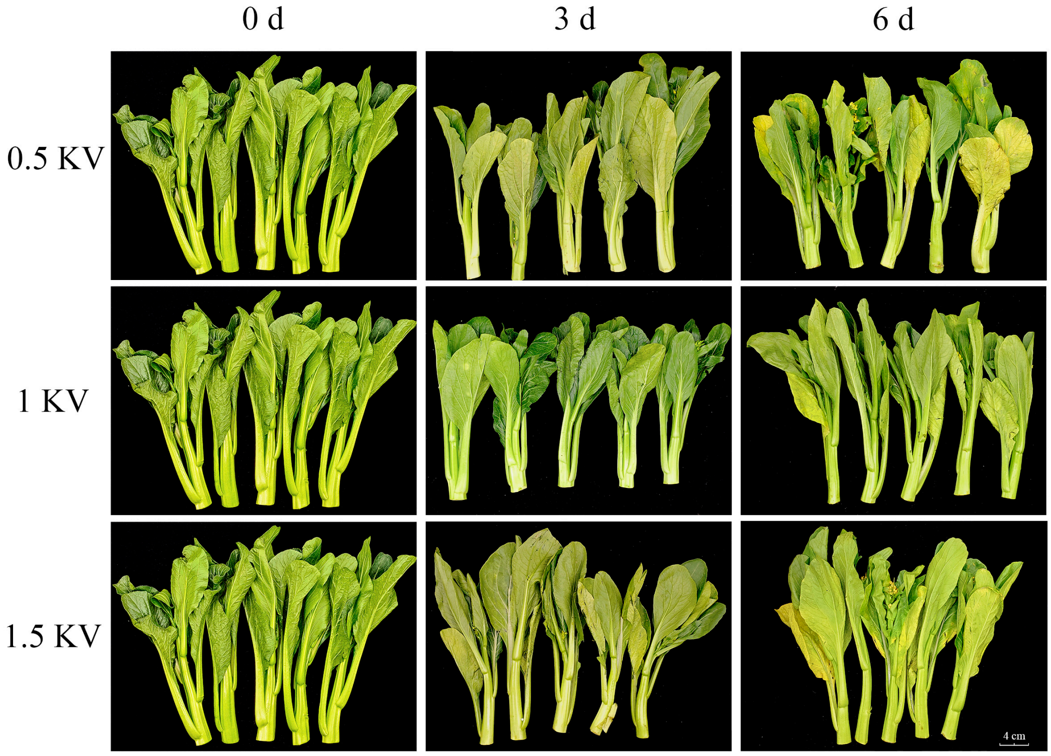

Figure S1. Preliminary observation of the effects of different LVEF intensities (0.5, 1, and 1.5 kV) combined with MAP on the visual quality of Chinese flowering cabbage during storage (0–6 days).

The images show representative samples stored under MAP combined with different LVEF intensities. At Day 6, samples treated with 1 kV maintained better visual quality and greener appearance compared with those treated with 0.5 kV and 1.5 kV, which exhibited varying degrees of yellowing.

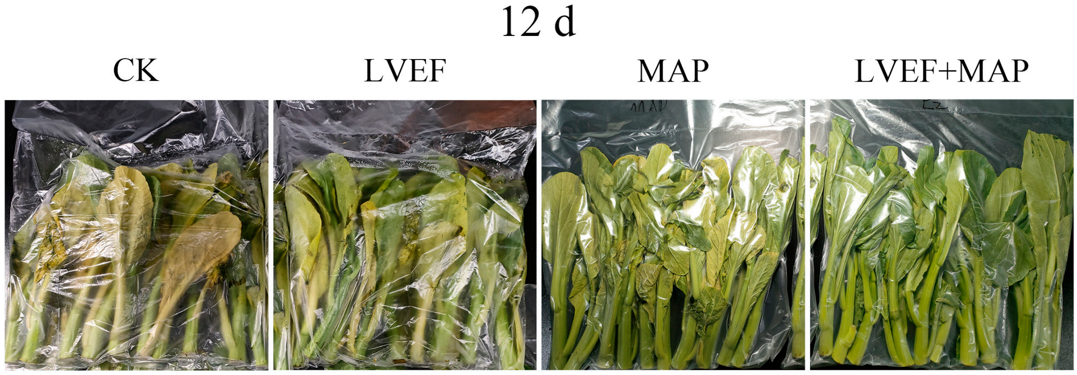

Figure S2. Visual representation of Chinese flowering cabbage samples in packaging bags under different treatments at 12 d of storage (CK, LVEF, MAP, and LVEF+MAP).
